# Study on the Optimum Cutting Parameters of an Aluminum Mold for Effective Bonding Strength of a PDMS Microfluidic Device

**DOI:** 10.3390/mi8080258

**Published:** 2017-08-22

**Authors:** Caffiyar Mohamed Yousuff, Mohd. Danish, Eric Tatt Wei Ho, Ismail Hussain Kamal Basha, Nor Hisham B. Hamid

**Affiliations:** 1 Department of Electrical and Electronics Engineering, Universiti Teknologi PETRONAS, Seri Iskandar 32610, Malaysia; hotattwei@utp.edu.my (E.T.W.H.); ismailhussain22@gmail.com (I.H.K.); 2 Department of Mechanical Engineering, Universiti Teknologi PETRONAS, Seri Iskandar 32610, Malaysia

**Keywords:** micro milling, microchannels, surface roughness, bonding strength

## Abstract

Master mold fabricated using micro milling is an easy way to develop the polydimethylsiloxane (PDMS) based microfluidic device. Achieving high-quality micro-milled surface is important for excellent bonding strength between PDMS and glass slide. The aim of our experiment is to study the optimal cutting parameters for micro milling an aluminum mold insert for the production of a fine resolution microstructure with the minimum surface roughness using conventional computer numerical control (CNC) machine systems; we also aim to measure the bonding strength of PDMS with different surface roughnesses. Response surface methodology was employed to optimize the cutting parameters in order to obtain high surface smoothness. The cutting parameters were demonstrated with the following combinations: 20,000 rpm spindle speed, 50 mm/min feed rate, depth of cut 5 µm with tool size 200 µm or less; this gives a fine resolution microstructure with the minimum surface roughness and strong bonding strength between PDMS–PDMS and PDMS–glass.

## 1. Introduction

Microfluidics is a popular technology for fluidic processing, chemical analysis and synthesis in a broad range of applications ranging from medical diagnostics and therapeutics [1], chemical processing [2], and food processing [3] to environmental monitoring [4]. Microfluidic devices are capable of precise and repeatable manipulations on nanoliter volumes of fluid and these capabilities are critically dependent on the geometry and dimensions of the microchannels. Devices with micrometer-sized channels impose physical forces on fluids to perform a variety of manipulations such as mixing, separation, insertion. 

A key attraction of microfluidics technology is that high precision devices can be rapidly fabricated at low cost. Although numerous fabrication methods exist [5,6,7], microfluidic chips are most easily fabricated by molding formable polymers, such as polydimethylsiloxane (PDMS), which are primarily fabricated for proof-of-concept microfluidic devices. To ensure correct operation on each fabricated device, microchannel geometry must be accurately imprinted from mold to polymer. The molds leave the extruded features which enables the inverse of the channel structures to be formed on polymer substrates (PDMS). Molds may be fabricated in silicon, glass, polymer or quart substrates by photo lithography [8]. Alternatively, molds may also be micro-machined from metal substrates such as copper, nickel, aluminum and brass [9,10,11,12,13,14,15,16]. Metal molds can be fabricated using conventional computer numerical control (CNC) machines and retain the accuracy of features over many more molding cycles compared to molds created by photo lithography because photoresist molds are relatively easily damaged [17] and have limited life cycles: 10 cast and peel off cycles in the case of the PDMS device [18] and <5 cycles in some cases such as hot embossing [19] with silicon as the substrate material; however, the SU8 mold pattern created over the thick copper substrate increased the molding cycles at least 40-fold [20], whereas the metal mold could retain the structure after a number of cycles [21].

Full three-dimensional channels are challenging to form by one-step molding. Instead, it is common practice to mold halves of the intended device bisected along the longitudinal section of the micro-channels. The molded parts are subsequently bonded to its complement shape or to a stiff planar substrate such as a glass slide [22]. Instead of adhesives, bonding is achieved by surface treatment of PDMS and glass via UV Ozone treatment [23] or by oxygen plasma treatment [24]. For PDMS surfaces that are treated with oxygen plasma or UV/ozone, the surface is made hydrophilic by replacing the surface methyl groups—bounded to the Si atom within the PDMS structure—with silanol groups (Si–OH). These new groups tend to chemically interact with other functional groups, allowing to selectively modify the surface and resulting in an effective bonding with the glass or PDMS itself. A strong bond is crucial for the microchannel to function well under high fluidic pressures. 

In this two-part assembly, the strength of the bonding is influenced by several factors: surface roughness [25,26,27,28], plasma exposure time [29,30,31] PDMS curing ratio [32], adhesive glue [33], and substrate material [34]. Among these factors, the roughness of the bonding surface is most crucial. In an earlier study, using the oxygen plasma treatment process for bonding, it was experimentally observed that the greater the plasma exposure time, the higher the surface roughness; this resulted in poor bonding [29]. Similarly, in the case of the Printed Circuit Board (PCB) mold, the rough surface in the PCB substrate affects the bonding strength of PDMS [27]. In this context, it was observed that the molds created by photo lithography have high surface smoothness because the development phase in this process removes all the photoresist outside the “inverse” channel region, thereby exposing the seed silicon layer which has a smooth surface and is appropriate for bonding. In contrast, metal micromachining is a bulk shaping process and casted PDMS will have the surface of the inverse master mold that is exposed after milling, thus high surface smoothness in metal molds is a crucial parameter for effective bonding.

The most commonly considered parameters which affect the surface roughness are cutting speed, feed rate and depth of cut. Nevertheless, Savage et al. [35] reported that the tool diameter should also be considered as a major contributing factor in the surface roughness recognition model. From experimental investigation, they studied that the tool size has a great impact on surface roughness and observed that smaller tool size with a higher feed rate has reduced surface roughness. Zhenyu Shi et al. [36] experimentally studied that the cutting tool geometry such as flute number and cutting edge radius are the influential parameters on surface topography. Lekkala et al. [37] experimentally studied that the depth of cut and the tool diameter are the main parameters which influence the burr height. They observed that the larger tool size has more burr formation than the smaller tool size which eventually increases the surface roughness of the work piece. Bajpai et al. [38] investigated experimentally that tool diameter and number of flutes contribute to the surface roughness of the machined work piece. It was observed that increasing tool diameter results in higher surface roughness although more flute numbers smoothen the surface. Amin et al. [39] studied the effect of cutter diameter on surface roughness on soda lime glass. They reported that the low surface roughness in the range of 0.08 μm is achieved using a 0.5 mm carbide end mill cutter. Based on the discussed literature, it was anticipated that smaller tool size is important to fabricate a good quality metal mold which will lead to the production of a highly smooth surface. In addition to this, it is also important to achieve a narrow channel gap between the two extruded regions which are necessary in microfluidic device fabrication and can only be obtained by using a smaller tool size as a bigger tool size will create a much wider channel than required.

In machinability studies, the statistical design of experiments has been used quite extensively. The statistical design of experiments refers to the process of planning the experiment so that the appropriate data can be analyzed by statistical methods, resulting in valid and objective conclusions. In order to establish an adequate functional relationship of a machinability model between the input of independent cutting variables (cutting speed, feed, depth of cut) and the output, known as responses (tool life, surface roughness, cutting force, material removal rate etc.) of a machining process, a large number of tests are needed for each and every combination of cutting tool and work-piece materials. This increases the total number of tests and as a result the experimentation cost also increases. Design and methods such as response surface methodology (RSM) [40] and Taguchi methods [41] are now widely used in place of a one-factor-at-a-time experimental approach which is time consuming and exorbitant in cost. RSM is a dynamic and foremost important tool of design of experiment (DOE), wherein the relationship between the output variables of a process with its input parameters (cutting conditions) is mapped to achieve the objective of maximization or minimization of the response properties. RSM saves cost and time by reducing the number of experiments required.

In this paper, the study of optimal cutting parameters to maximize surface smoothness of micro machined aluminum molds for microfluidic devices to achieve high bonding strength was reported. The RSM was applied to identify the best combination of cutting speed, feed rate, depth of cut and tool size.

## 2. Materials and Methods 

### 2.1. Experiment Design and Fabrication of Aluminium Master Mold

**Figure 1:**
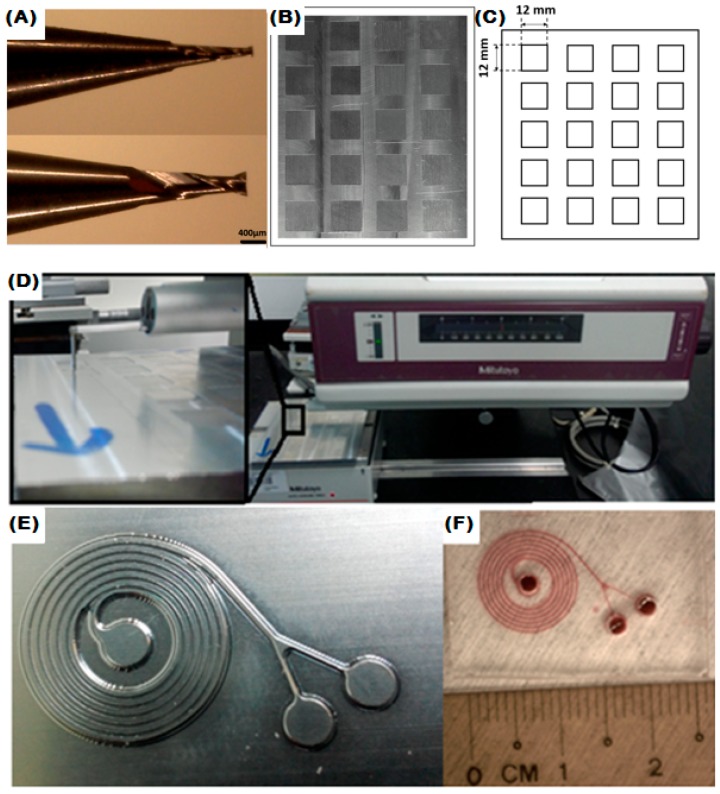
(**A**) Micro Endmill tool; (**B**) Micro-milled chamber with different cutting parameters; (**C**) Schematic of the micromilling chamber; (**D**) Surface Roughness Tester with Zoom out view; (**E**) Spiral master mold with optimum cutting combination; (**F**) Polydimethylsiloxane (PDMS) device fabricated from the master mold.

Endmilling was performed on aluminum substrate (Al6061 alloy, Alcan Aluminium Limited, Montreal, QC, Canada) using two tungsten carbide micro endmills of 200 µm and 400 µm size (Kriss Precision, Penang, Malaysia), shown in Figure 1A, on a 5-axis CNC machine (Mazak Variaxis 630 5x, Yamazaki Mazak Singapore Pte Ltd., Jurong, Singapore). The aluminum block of size 75 mm × 75 mm was tightly fixed on the platform to maintain the stability while milling. The aluminum block has 20 reservoirs, each reservoir of size 12 mm × 12 mm is micro-milled with a variable cutting parameter, shown in Figure 1B and schematic of the micromilling chamber is shown in Figure 1C. To measure the roughness of the micro milled Aluminum substrate with various cutting parameters, Surftest SV-3000 CNC Ultra-high Precision Surface Roughness Tester (Mitutoyo Corporation, Takatsu-ku, Kawasaki, Kanagawa, Japan) was used at a different position inside a reservoir, shown in Figure 1D. Finally, a master mold fabricated with optimized cutting parameters is shown in Figure 1E.

### 2.2. Experimental Setup

To understand the influence of varying parameters such as tool size (T), spindle speed (N), depth of cut (DOC), and the feed rate (F) on the micro milled surface quality of an aluminum mold insert, various factor levels, listed in Table 1, were used in the experiments. Each set of experiments was conducted using new end mill cutters to avoid any effect of the tool wear on the results. RSM was employed to design the experiments and to analyze the results. The full experimentation scheme with responses is discussed in the results and discussion section.

### 2.3. Fabrication of PDMS Microfluidic Device

Next, the polydimethylsiloxane (PDMS, Sylgard 184, Dow Corning, Midland, MI, USA) device was fabricated from the aluminum mold to test the bonding strength between PDMS–PDMS and PDMS–glass for different surface roughnesses. PDMS was mixed to a 1:10 ratio between curing agent and PDMS monomers. The master mold was placed inside an acrylic holder to remove the PDMS easily, thus not damaging the mold structure. The mixture was poured over the aluminum master, degassed for 30 min in the open atmosphere, and cured at 70 °C in an oven for 3 h. PDMS is carefully peeled off from the mold. The inlets are punched using a 2 mm biopsy punch. The PDMS device was cleaned using scotch tape to remove any dust particles on the surface, then cleaned with ethanol. After that, the device was permanently bonded to the glass slide using oxygen plasma treatment (Harrick Plasma, Ithaca, NY, USA) and UV ozone system (Nova scan Inc., Ames, IA, USA) for 3–5 min. The plasma- and UV-treated device is immediately baked on a hot plate at 80 °C for 15 min in order to increase the bonding strength. The spiral PDMS microfluidic device after bonding is shown in Figure 1F.

### 2.4. Bonding Strength Measurements

**Figure 2:**
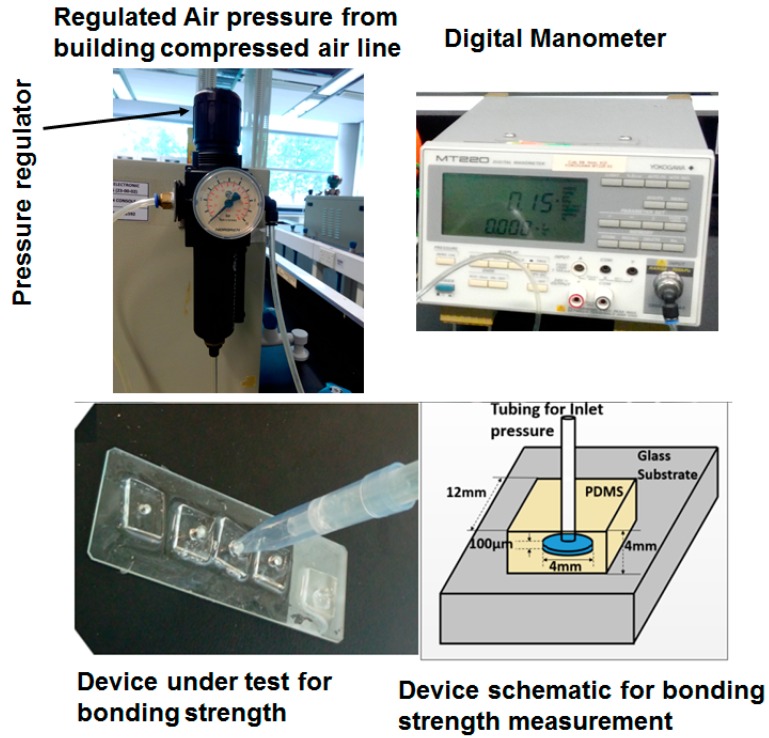
Setup for measuring bonding strength using the building compressed air line and digital manometer.

In the next step, the bonding strength was measured using a Yokogawa MT 220 Digital manometer (Yokogawa Meters & Instruments Corporation, Tokyo, Japan), at various pressures between 0 to 500 kPa through the inlet tubing hole. The regulated air pressure from the building compressed air line was used for inlet pressure. The setup for measuring bonding strength is shown in Figure 2. The effect of various pressures on bonding strength was tested for the casted PDMS from micro milled surface having minimum and maximum surface roughness obtained from 200 µm and 400 µm tool size. 

## 3. Results and Discussion

Response surface methodology was used to analyze the impact of the three cutting parameters on the surface roughness of the aluminum mold. The cutting parameters that have been investigated in the present study are spindle speed (N), depth of cut (DOC) and feed rate (F) for two different tools.

### 3.1. Experimental Design with Experimental Responses

The design of experiment (DoE) employed in the current study is the standard center composite design (CCD) for studying the process. The CCD used here has all the star points at the center of the face of the cube which corresponds to the value of α = 1 [42]. This type of CCD is referred to as face centered CCD [43]. The center points were repeated six times. A full quadratic polynomial was used to predict the fitting model considering all factors of the response surface. Table 2 shows the whole experimental scheme as designed here along with the experimental results. The surface roughness value was found in the range of 0.169 µm to 0.274 µm and 0.290 µm to 0.389 µm for the tool T1 and T2 respectively.

### 3.2. Regression Equations 

Analysis of variance (ANOVA) was conducted by applying F-test. If the *p*-value was less than 0.05, then it was considered that the model or the parameter under consideration was significant and insignificant otherwise. After conducting ANOVA for the present model for T1, the *p*-value for the whole model turns out to be less than 0.05 which is also shown in Table 3. This implies that the model is significant. The *p*-value for lack of fit of the model was greater than 0.05 which suggests that the model was appropriate. 

The resulting model is shown in Equation (1).

(1) $$\mathrm{Surface}\;\mathrm{Roughness}=+0.18667+4.72727\times 10^{-8}\times A+9.74818\times 10^{-4}\times B-\;1.29182\times 10^{-3}\times C-1.70909\times 10^{-10}\times A^{2}-2.10909\times 10^{-6}\times B^{2}\;+8.90909\times 10^{-5}\times C^{2}+1.00\times 10^{-9}\times \mathrm{AB}+7\times 10^{-8}\times \mathrm{AC}-2\times 10^{-6}\times \mathrm{BC}$$

The *p*-value also gives the significance of a particular factor for the model. In the present model, out of nine factors (spindle speed, feed rate, depth of cut, square of each parameter and product of each parameter taking two at a time) five have a *p*-value less than 0.05 which indicates their significance. In this case, those five parameters are spindle speed, feed rate, depth of cut and square of spindle speed and square of the feed rate. The remaining parameters of the model can be eliminated by following the backward elimination method and the new model is shown by Equation (2).

(2) $$\mathrm{Surface}\;\mathrm{Roughness}=+0.17890-1.55\times 10^{-7}\times A+9.03\times 10^{-4}\times B+1.34\times \;10^{-3}\times C-1.375\times 10^{-10}\times A^{2}-1.775\times 10^{-6}\times B^{2}$$

Following the same procedure for the tool T2, the ANOVA table for the model is shown in Table 4. Here, the *p*-value for the model and the lack of fit were 0.0012 and 0.01787 respectively, suggesting that the model is significant and appropriate for the analysis.

After eliminating the insignificant parameters from the model by the backward elimination method, the final model that can predict surface roughness is shown by Equation (3).

(3) $$\mathrm{Surface}\;\mathrm{Roughness}=+0.2911-2.4\times 10^{-6}\times A+1.474\times 10^{-3}\times B-4.96\times 10^{-6}\times B^{2}$$

### 3.3. Adequacy of the Model

**Figure 3:**
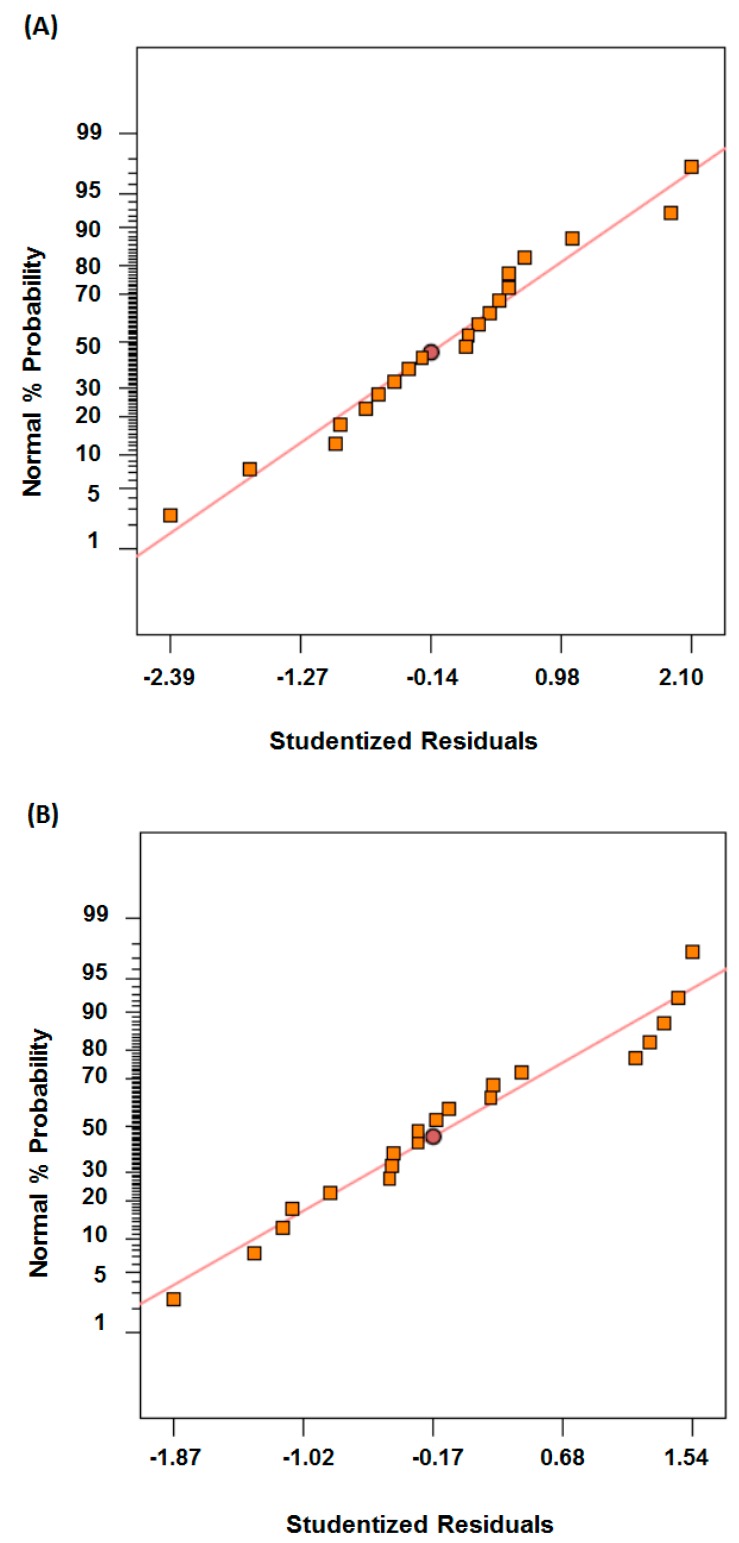
Plot of normal probability vs. studentized residuals (**A**) For tool diameter, T1; (**B**) For tool diameter, T2.

The adequacy of the model can be analyzed by plotting normal probability and studentized residuals. Figure 3 shows the plot of normal probability and studentized residuals. It is evident from Figure 3 that the residuals follow a straight line and there is no particular trend of the residuals in both cases which justifies the adequacy of the model. Therefore, these models can be used to predict the response or optimize the parameters for the response.

### 3.4. 3D Plots for Surface Roughness

**Figure 4:**
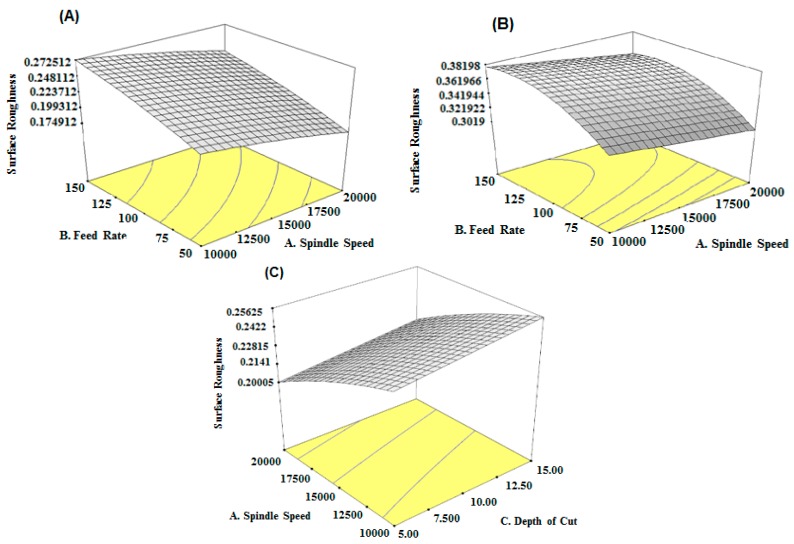
Three-dimensional plot of surface roughness vs. feed rate and spindle speed (**A**) For tool diameter; T1 (**B**) For tool diameter, T2; (**C**) Surface roughness vs. spindle speed and depth of cut for T1.

The 3D plots for the model, shown in Figure 4A,B, predict that, for both tool T1 and T2, the feed rate has the greatest influence on the surface roughness of the micromachined aluminium followed by spindle speed and depth of cut. It is observed from Figure 4C, depth of cut has very least effect with spindle speed for tool T1 whereas, from Equation (3), the model predicts that, depth of cut has very negligible effect with tool T2 and thus not included in the model. 

While machining with tool T1, incrementing the spindle speed and decreasing the feed rate results in decreased surface roughness. Similar trends were also seen for tool T2; the only difference is in the magnitude of the surface roughness value. Milling with a smaller tool diameter produces high surface smoothness when compared to milling with a bigger tool diameter for the same set of parameters. A possible reason for this could be that larger tools produce large chips and give rise to larger burr height. On the other hand, smaller tools provide the least metal removal and therefore produce little burr. 

This increased burr size is the cause of the increased surface roughness in a larger tool compared to a smaller tool [38,44].

### 3.5. Verification Test

**Figure 5:**
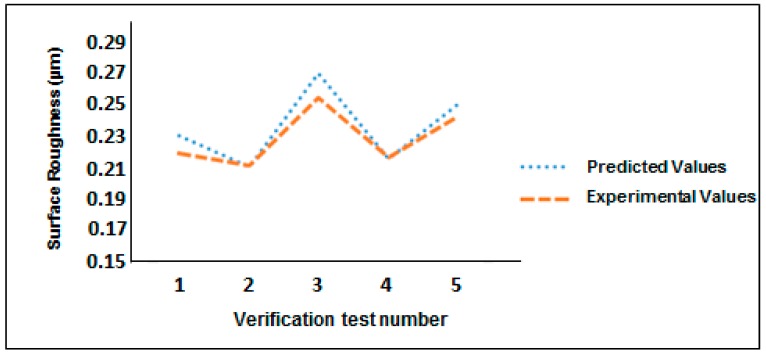
Verification test with random cutting parameters.

A verification test was performed on five different samples for five random cutting parameters analytically obtained with the tool diameter T1 as T1 provides an enhanced surface finish compared to tool T2. Surface roughness was noted at five different positions on each sample after machining and the average of those five readings was considered as a response. Table 5 shows the results of the verification test with different cutting parameters and surface roughness (Ra). Figure 5 shows a graph of surface roughness with the verification test number. It is evident from Figure 5 that the model is reliable and can predict the surface roughness of the machined aluminum mold insert. The percentage error in the predicted and measured value of the surface roughness was found to be 0.4% to 5.92%.

### 3.6. Optimization of the Cutting Parameters

Optimization of cutting parameters during milling of aluminium mold was done to obtain the minimum value of the response (surface roughness) with highest importance. As tool T1 has the minimum surface roughness in contrast to tool T2, the optimization result for tool T1 alone was carried out. The cutting parameters were assigned a value in the range between the lowest and highest value used during the experiments. Table 6 shows the goal and the ranges assigned to the response and cutting parameters.

The optimization results considering the goals presented in Table 6 with their desirability of occurrence for the diameter of tool T1 are shown in Table 7.

## 4. Effect of Tool Diameter on Microstructures

**Figure 6:**
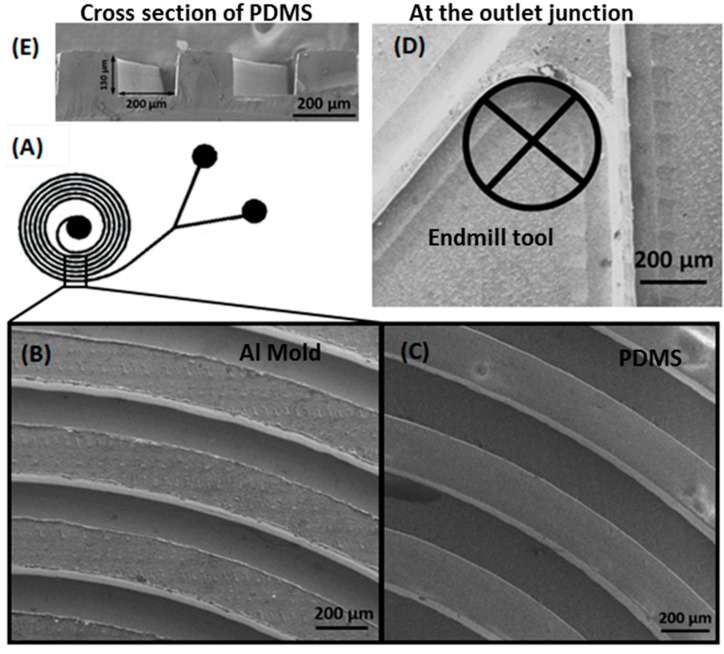
(**A**) Schematic of Spiral structure; (**B**) SEM micrograph of the aluminium mold; (**C**) SEM micrograph of the casted PDMS channel from the mold; (**D**) Outlet intersecting junction; (**E**) Cross section image of the PDMS channel.

Apart from the minimum surface roughness, it is possible to achieve minimum curvature at the intersecting junction and minimum gap between the two extruded structures using a smaller tool diameter. The schematic of our fabricated spiral structure is shown in Figure 6A. The SEM micrograph of the aluminium master mold with a minimum channel gap of 200 µm, is shown in Figure 6B; curvature at the outlet intersecting junction with 200 µm diameter, which is performed using a 200 µm end mill cutter, is shown in Figure 6D. It was also observed that the casted PDMS channel from the aluminium mold is quite smooth, as shown in Figure 6C. The cross section of the PDMS channel is a clear rectangular channel, shown in Figure 6E which is much closer to the PDMS casted from the SU8 mold. The small round corners and small gaps between the channels are detrimental factors in many applications. This fine resolution structure would not be possible with a bigger tool size.

## 5. Bonding Strength Measurement

The bonding strength between PDMS to PDMS and PDMS to glass is characterized by the different surface roughnesses of the aluminum mold. Bonding strength of PDMS with minimum and maximum surface roughness obtained from the tool sizes T1 and T2 were tested in order to understand their effect on bonding. It was observed that, with the standard 10:1 PDMS mixing ratio, the bonding strength from 270 to 314 kPa is achieved with the minimum surface roughness and 185 to 270 kPa is achieved with the maximum surface roughness with possible standard bonding methods for PDMS as shown in Table 8. From Table 8, it is shown that the lower the surface roughness, the greater the bonding strength which could be achieved with smaller tool bits. These bonding strengths are comparable to the bonding strength reported in the literature [31,45,46] for both a lithography-based and metal-based mold. Though PDMS adhesive and partially cured PDMS have a bonding strength of over 500 kPa, they are more prone to channel blockages and device failure.

## 6. Conclusions

The aim of this study was to explore the optimal parameters, using the RSM method, in micromachining an aluminum mold insert for the fabrication of a PDMS microfluidic device. It was observed that the cutting parameters have a significant effect on the surface roughness of the aluminum mold. Additionally, the influence of surface roughness on the bonding strength between PDMS and glass was analyzed. From this study, the following main conclusion can be drawn:

(1) The feed rate has the greatest influence on the surface roughness followed by the spindle speed for both tools: 200 µm and 400 µm. Depth of cut has the least effect in the case of smaller tool size and almost no effect was observed for the larger tool size.
(2) From the roughness measurement, the samples with tool T1 had lower roughness compared to tool T2.
(3) With RSM factor analysis, the optimal cutting parameters were found to be as follows: 20,000 rpm spindle speed, 50 mm/min feed rate, depth of cut 5 µm with tool size 200 µm or less. These parameters are the best choice to achieve a smooth micro-milled surface and fine resolution structure using a conventional CNC machine.
(4) For the given cutting combinations, a minimum surface roughness of 0.169 µm is achieved; this provides efficient PDMS bonding strength of greater than 300 kPa.
(5) It is also observed that a much narrower gap between the structures can be produced by using the smallest tool diameter with a fine resolution microstructure at a reduced time; this is on par with the photo lithography-based microstructure without the requirement of a clean room.

## Figures and Tables

**Table 1:** Various levels of four factors, including spindle speed, feed rate, and depth of cut, used in the experiments.

Notation	Factor/Level	Tool Diameter (T1) 200 µm			Tool Diameter (T2) 400 µm		
		−1	0	1	−1	0	1
A	Spindle speed (rpm)	10,000	15,000	20,000	10,000	15,000	20,000
B	Feed rate (mm/min)	50	100	150	50	100	150
C	Depth of cut (µm)	5	10	15	5	10	15

**Table 2:** Experimental results for surface roughness with different tools.

Run No.	Spindle Speed (rpm)	Feed Rate (mm/min)	Depth of Cut (µm)	Surface Roughness for T1 (µm)	Surface Roughness for T2 (µm)
1	15,000	100	10	0.270	0.371
2	15,000	100	10	0.203	0.369
3	20,000	150	5	0.225	0.340
4	20,000	50	5	0.169	0.388
5	15,000	100	5	0.231	0.319
6	15,000	100	10	0.253	0.371
7	15,000	150	10	0.221	0.320
8	20,000	50	15	0.251	0.359
9	15000	100	15	0.199	0.370
10	10,000	150	5	0.233	0.333
11	20,000	150	15	0.241	0.309
12	15,000	100	10	0.232	0.364
13	10,000	150	15	0.182	0.368
14	15,000	100	10	0.274	0.389
15	20,000	100	10	0.230	0.355
16	10,000	50	5	0.229	0.350
17	15,000	100	10	0.233	0.320
18	10,000	50	15	0.210	0.367
19	15,000	50	10	0.226	0.341
20	10,000	100	10	0.241	0.351

**Table 3:** Analysis of variance (ANOVA) table for T1.

Source	Sum of Squares	Degree of Freedom	Mean Square	F-Value	*p*-Value (Prob > F)
Model	0.013	9	1.425 × 10^−3^	195.90	<0.0001
A	4.580 × 10^−3^	1	4.580 × 10^−3^	629.54	<0.0001
B	7.508 × 10^−3^	1	7.508 × 10^−3^	1032.04	<0.0001
C	4.489 × 10^−4^	1	4.489 × 10^−4^	61.71	<0.0001
A^2^	5.020 × 10^−5^	1	5.020 × 10^−5^	6.90	0.0253
B^2^	7.645 × 10^−5^	1	7.645 × 10^−5^	10.51	0.0088
C^2^	1.364 × 10^−5^	1	1.364 × 10^−5^	1.88	0.2008
AB	5.000 × 10^−7^	1	5.000 × 10^−7^	0.069	0.7985
AC	2.450 × 10^−5^	1	2.450 × 10^−5^	3.37	0.0964
BC	2.000 × 10^−6^	1	2.000 × 10^−6^	0.27	0.6115
Residual	7.275 × 10^−5^	10	7.275 × 10^−6^	-	-
Lack of Fit	5.941 × 10^−5^	5	1.188 × 10^−5^	4.46	0.0634
Pure Error	1.333 × 10^−5^	5	2.667 × 10^−6^	-	-

**Table 4:** ANOVA table for T2.

Source	Sum of Squares	Degree of Freedom	Mean Square	F-Value	*p*-Value (Prob > F)
Model	8.135 × 10^−3^	9	9.039 × 10^−4^	8.54	0.0012
A	1.440 × 10^−3^	1	1.440 × 10^−3^	13.60	0.0042
B	5.808 × 10^−3^	1	5.808 × 10^−3^	54.86	<0.0001
C	4.840 × 10^−5^	1	4.840 × 10^−5^	0.46	0.5143
A^2^	1.642 × 10^−6^	1	1.642 × 10^−6^	0.016	0.9034
B^2^	4.845 × 10^−4^	1	4.845 × 10^−4^	4.58	0.0581
C^2^	1.364 × 10^−5^	1	1.364 × 10^−5^	0.13	0.7271
AB	4.500 × 10^−6^	1	4.500 × 10^−6^	0.043	0.8408
AC	2.000 × 10^−6^	1	2.000 × 10^−6^	0.019	0.8934
BC	5.000 × 10^−5^	1	5.000 × 10^−5^	0.47	0.5076
Residual	1.059 × 10^−3^	10	1.059 × 10^−4^	-	-
Lack of Fit	7.479 × 10^−4^	5	1.496 × 10^−4^	2.41	0.1787
Pure Error	3.108 × 10^−4^	5	6.217 × 10^−5^	-	-

**Table 5:** Verification test.

Test No.	Spindle Speed (rpm)	Feed Rate (mm/min)	Depth of Cut (µm)	Tool	Predicted Value of Ra (µm)	Experimental Value of Ra (µm)
1	20,000	100	10	T1	0.230	0.219
2	10,000	50	15	T1	0.210	0.211
3	15,000	100	10	T1	0.270	0.254
4	20,000	150	05	T1	0.215	0.216
5	20,000	50	15	T1	0.250	0.242

**Table 6:** Goals and ranges assigned to the response and cutting parameters.

Response/Cutting Parameters	Goal	Lowest Value	Highest Value
Surface roughness (µm)	Minimum	-	-
Spindle speed (rpm)	In the range	10,000	20,000
Feed rate (mm/min)	In the range	50	150
Depth of cut (µm)	In the range	5	15

**Table 7:** Optimization results for the surface roughness using the diameter of tool T1 (200 µm).

Serial No.	Spindle Speed (rpm)	Feed Rate (mm/min)	Depth of Cut (µm)	Surface Roughness (µm) T1	Desirability
1	19,889.42	50.01	5.10	0.168983	1.000
2	19,999.97	51.21	5.00	0.169089	0.999
3	19,837.82	50.06	5.00	0.169169	0.998
4	20,000.00	50.00	5.74	0.169201	0.998
5	19,763.74	50.01	5.00	0.169552	0.993

**Table 8:** Comparison of surface roughness with bonding strength using various bonding techniques.

Surface Roughness (µm)	Bonding Strength (kPa)				
	Oxygen Plasma Treatment		UV-Ozone Treatment		PDMS Adhesive and Partially Cured PDMS
	PDMS–Glass	PDMS–PDMS	PDMS–Glass	PDMS–PDMS	PDMS–PDMS and PDMS–Glass
0.17	270 ± 20	180 ± 20	314 ± 20	95 ± 20	>500 kPa
0.27	263 ± 20	168 ± 20	301 ± 20	92 ± 20	>500 kPa
0.32	228 ± 20	147 ± 20	288 ± 20	92 ± 20	>500 kPa
0.4	185 ± 20	105 ± 30	270 ± 20	87 ± 20	>500 kPa
